# Impact of the landfill of ashes from the smelter on the soil environment: case study from the South Poland, Europe

**DOI:** 10.1007/s10653-019-00435-y

**Published:** 2019-10-08

**Authors:** Anna Twaróg, Magdalena Mamak, Henryk Sechman, Piotr Rusiniak, Ewelina Kasprzak, Krzysztof Stanek

**Affiliations:** Faculty of Geology, Geophysics and Environmental Protection, AGH University of Science and Technology, al. A. Mickiewicza 30, 30-059 Kraków, Poland

**Keywords:** Ash landfill, Metallurgical ash dump, Heavy metals, Soil environment, CO_2_ flux, Impact of industry

## Abstract

The following research describes the influence of a metallurgical ash dump on both the soil environment and the atmosphere. Soil samples were collected along a line positioned on an unprotected, hazardous ash dump and extended into the adjacent, arable land. Three soil depths were sampled at 0–20-, 20–40- and 40–60-cm depth intervals, and in each sample, pseudo-total concentrations of Cd, Cr, Cu, Fe, Mn, Ni, Pb, Ti, Zn, Li, Sr and V were analyzed. Additionally, emissions of CH_4_ and CO_2_ were measured at each sampling site. All emission measurements were taken in the same day, and the duration of gas measurements in each place was six minutes. The results demonstrate elevated concentrations of Cu, Cr, Pb and Zn on dump surface and along its margins, where the maximum concentrations of these elements are, respectively, 82, 23, 1144 and 8349 mg kg^−1^. Obtained results exceed several times both the natural background values and the values typical of local soils in the southern Poland. Moreover, natural background values for Fe, Mn, Ni, Li, Sr and V were exceeded, as well. Along the sampling line, no methane emission was detected, whereas the carbon dioxide flux varied from 7 to 42 g m^−2^ d^−1^. The reconnaissance study of the ash dump revealed a high contamination level of soils with heavy metals, which, together with the changes of soil environment, may cause migration of pollutants into the adjacent areas and, consequently, may generate hazard to the environment and, particularly, to the living organisms. Hence, further studies are necessary in order to evaluate the soil quality and the leaching of heavy metals from the dump.

## Introduction

Operation of mining and metallurgical plants within the urban areas may significantly affect the quality of both the soil and the aquatic environments and, indirectly, may influence also the human health and life (Janas and Zawadzka 2017). The recent studies carried on around smelters, e.g., in France (Douay et al. 2008; Grumiaux et al. 2015), Uzbekistan (Shukurov et al. 2014), China (Li et al. 2011) and Moldova (Stafilov et al. 2010), revealed that industrial activity results in an uncontrolled formation of zones polluted with trace elements. Industrial wastes are generated at any stages of technological processes, i.e., during the production, storage and transport, and then are disposed at dumps (Janas and Zawadzka 2017). Apart from hazards caused by emission of trace elements from industrial plants (e.g., smelters) into the atmosphere (Shukurov et al. 2014; Grumiaux et al. 2015), environmental pollution appears also in the vicinity of designed but uncontrolled industrial waste dumps (Kasassi et al. 2008; Grabas 2009; Klojzy-Karczmarczyk and Mazurek 2009; Gowd et al. 2010; Karczewska and Kabala 2010). Such objects are particularly hazardous to the environment due to still inefficient protection technologies against their negative environmental effects (Aderemi et al. 2011; Janas and Zawadzka 2017). Additional controls of potential migration of pollutants around the waste dumps come from climatic conditions, land morphology and applied protection measures (e.g., barriers) (Janas and Zawadzka 2017).

Mechanisms of migrations of trace elements in the soil and aquatic environment are well known from other studies (Dube et al. 2001; Nowińska and Adamczyk 2013; Danila and Vasarevičius 2017). Heavy metals are accumulated in the top soil and then transported by plants to the food chain (Douay et al. 2008). In the hypergenic zone, mobility of heavy metals depends on the elements form and environmental conditions. Physicochemical factors controlling the mobility of elements include: water chemical composition, pH, Eh and the presence of particular chemical components (Nowińska and Adamczyk 2013). Generally, the mobility of heavy metals in soil increases as the pH decreases. The earlier studies show that mobility of Cd and Zn increases when the pH drops to below 6–6.5 (Gębski 1998), whereas Cu and Pb show similar properties below pH < 5 (Sady and Smoleń 2004).

In the southern Poland, agricultural soils are characterized by elevated concentrations of heavy metals (Terelak et al. 2000). Many authors found increased concentrations of Cu, Cd, Pb and Zn in soils from the southern Poland, originated from emission by metallurgical and mining plants in the region (Gorlach 1995; Rogóż 2003; Koncewicz-Baran and Gondek 2010). One source of potential pollutant in the region is unprotected waste dump of ashes originated from smelter.

The following research aims to evaluate the impact of an industrial waste dump localized in and urban area in Poland on both the soil environment and the atmosphere. We expect that elements are migrating from waste dump to the neighbor area because metals can be mobile. Authors also believed that oxidized forms of carbon from the ashes may increase carbon dioxide emission from contaminated soils, whereas increased activity of microorganisms in mixed layers of ash and hummus can produce additional amounts of greenhouse gases (CH_4_ and CO_2_).

## Characterization of the site

**Fig. 1:**
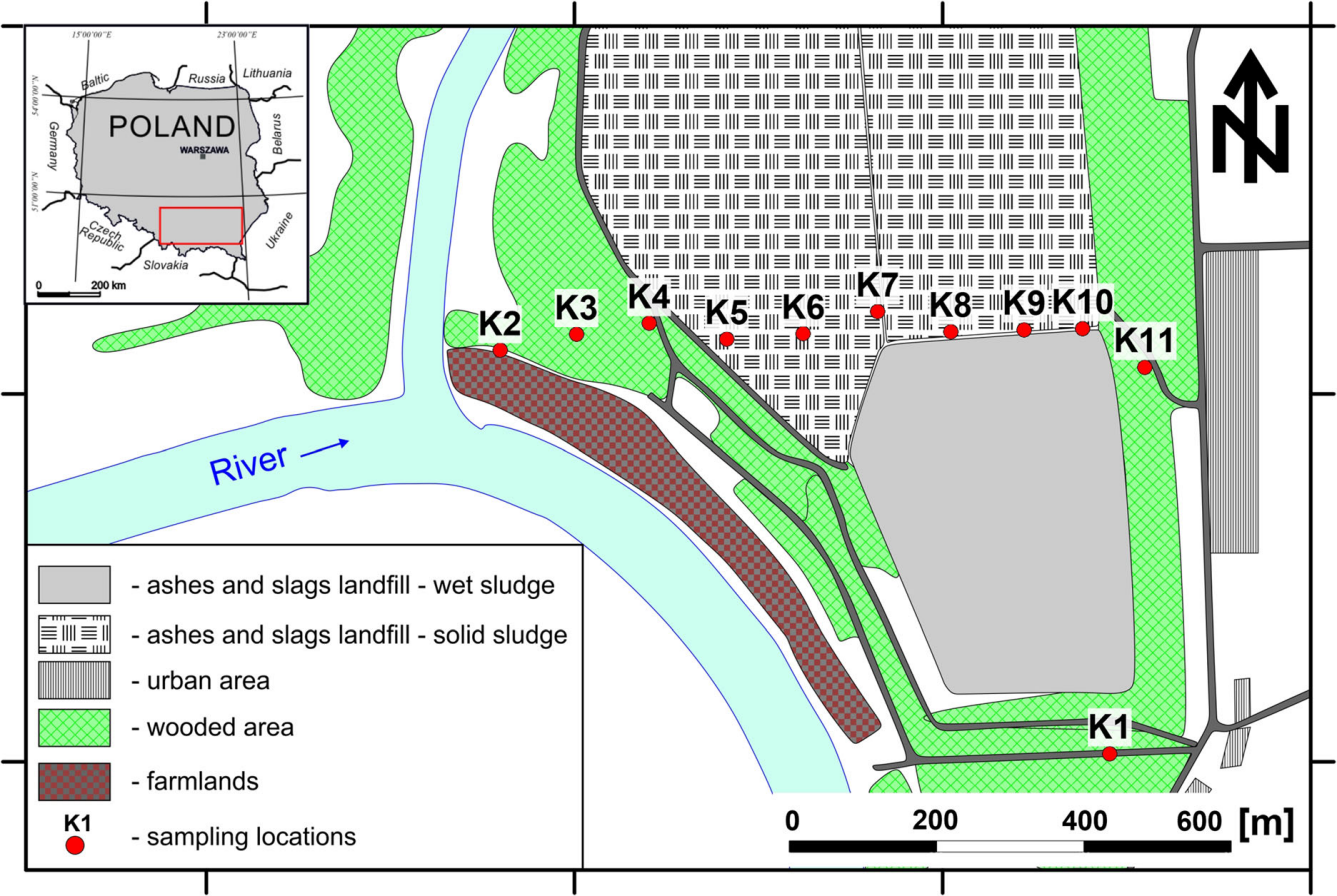
Localization map of sampling points and bounders of landfill

The study area is an industrial waste dump located within the area of one of the cities in southern Poland (Fig. 1). From the west, the disposal borders the river bend, whereas from the southwest and the east it neighbors the farmlands (crops include carrots, colza and courgettes). Moreover, from all sides, the disposal is surrounded by clusters of shrubs and trees. From both the south and the east of the dump, the busy communication routes occur together with the loopway of municipal transport line. The residential area is distant by about 200 m to the south from the dump, and the smelter, which is the owner of the dump, is located some 1.5 km to the north.

The dump was established in the mid-twentieth century. It includes three different sectors: ash and slag dump, ferruginous sludge dump and miscellaneous sludge dump. Despite the technological wastes (Grzesik 2007), the settling ponds collect also the final sludge from the sewage treatment plant of the smelter.

For the purpose of this research project, samples were collected from the settling ponds of ashes and slags (K5–K10) as well as from the cropfields, woodlands and barren lands (K1, K2, K3, K4 and K11) located in the close vicinity of the ponds (Fig. 1).

**Fig. 2:**
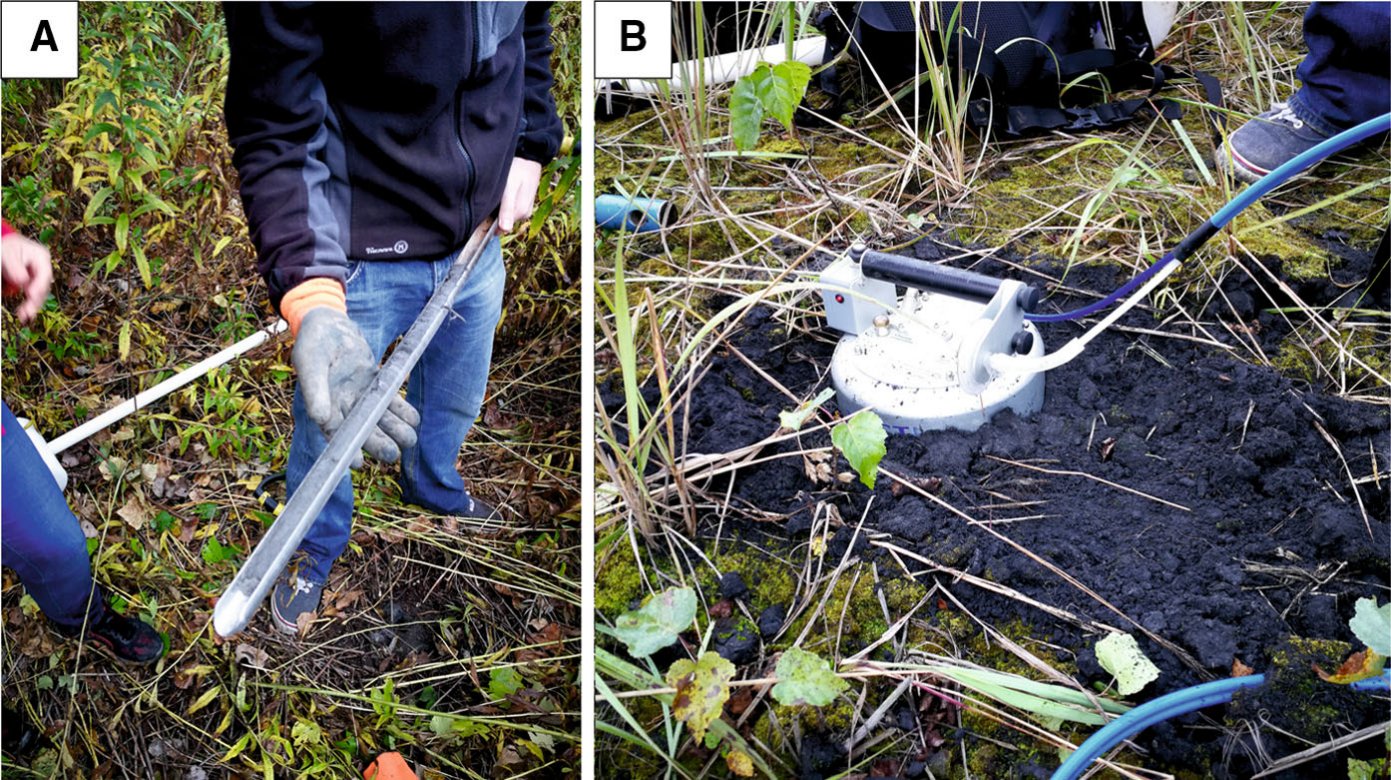
Collection of soil samples using the Egner’s stick (**a**) and measurement of gases emission using static chamber method (**b**)

The dump area is fenceless, which may cause hazard for humans not knowing that in these place there is a landfill of ashes contaminated with heavy metals. From the south (i.e., along the riverside), no information boards exist. Sparse boards are located only along the eastern border of the dump, along the main road, but they are obscured by shrubs (Fig. 1). Within the dump area, we found places where sludge covers vegetation (trees and shrubs) with a layer up to several tens of centimeters thick. The ash is present in the soil profile even as far as the K3 sampling site (Figs. 1, 8). Within the dump area, vegetation is mostly the sparse grass and moss, both growing onto the thick layer of dark ash (Fig. 2b). Additionally, the pollutants may seep from the dump to groundwaters and then may migrate toward the housing estates and the river.

## Methodology

### Collection of soil samples

Soil samples were collected at 11 sampling sites using the Egner’s stick (Fig. 2a). The K1 sample was taken at the site some 600 m distant from the sampling line, outside the disposal area. This is a reference sample, which provides the geochemical background for the study area. The remaining sampling sites were positioned at 100-m spacing along the sampling line cutting through the neutral zone (at both ends of the line) and the ash/slug sector (Fig. 1). At each sampling site, three soil samples were collected from three depth intervals: 0–20, 20–40 and 40–60 cm, into the tight polyethylene bags and transported to the laboratory. Additionally, at each sampling site, the soil profile was described together with local relief, atmospheric conditions (pressure and temperatures) as well as the GPS coordinates were recorded with the Garmin 650t device.

### Measurements of greenhouse gases emission

The flux of greenhouse gases (CH_4_ and CO_2_) from the soil to the atmosphere was measured with the static chambers method using the portable fluxmeter (Etiope et al. 2011; Twaróg et al. 2016; Baciu et al. 2018). This device is equipped with two detectors: WS–CH_4_-TLD for methane and LI820 for carbon dioxide, and enables the researcher to measure and record the changes in time of concentrations of gases filling the static chamber. The measurements of CH_4_ and CO_2_ emissions were executed directly at the soil sampling sites (Fig. 1).

### Sample preparation for analyses

Soil samples were dried at the room temperature and sieved using the mesh 1-mm sieve, in order to remove coarser fractions and plant remnants. Then, fraction < 1 mm was ground in an agate mortar and homogenized.

After mass reduction and averaging, the analytical samples were prepared for measurements of selected physical and chemical parameters including pH in distilled H_2_O with the potentiometric method, in accordance with the Polish Standard PN-ISO 10390:1997 (PN-ISO 1997) and the contents of organic matter, and carbonates were determined using the weight loss method, in which samples were ashed in a muffle furnace at 550 °C and 950 °C until the constant mass was obtained.

The main goal was to determine total concentration of selected heavy metals in soil samples after dissolution of non-silicate phases with *aqua regia* (HNO_3_:HCl, 1:3 ratio) at 130 °C in a DigiPREP HT mineralizer produced by SCP Science, in accordance with the standard ISO 11466:1995 (ISO 1995).

### Chemical analyses

The samples were analyzed with the use of inductively coupled plasma optical emission spectrometry (ICP-OES) Optima 7300DV PerkinElmer in accordance with ISO 11885:2007 (2007). Laboratory has implemented quality control system and regularly takes participation in the proficiency tests/interlaboratory comparisons (PT/ILC) with the satisfactory results (absolute Z-score lower than 2). The research included analysis blank laboratory samples (deionized water (18.2 MΩ cm) obtained by Milli-Q system) (Millipore, Bedford, MA), standard solution with certified value (ICP multielement standard solution VI Certipur^®^) and control sample with known environmental matrix. The working solutions were prepared by dilution with HNO_3_-acidified deionized water as needed. The quantification limits (LQ) for the elements determined were as follows: Li—0.005 mg/L, Cu, Mn, V—0.05 mg/L, Cd, Cr, Fe, Pb, Zn—0.1 mg/L, Sr, Ti—0.2 mg/L and Ni—0.5 mg/L. The LQ values include the dilution of the samples. The ICP-OES apparatus works with the radiofrequency (RF) power 1300 W. Nebulizer flow is set up as 0.8 L/min and plasma flow 15 L/min. Plasma viewing was axially/radially dependent on the element determined. Each analysis includes three replicated measurements. Processing mode of the results is a peak area with a 2-point manual background correction.

### Statistical data processing

The total contents of analyzed elements in soil samples were referred to the natural background values for the environment (after Kabata-Pendias and Pendias 1993) and to the permissible concentrations for the Soil Quality Group III (woodlands and shrubs, barren lands and various areas), in accordance with the *2016.09.01 Regulation of the Minister of Environment on the method for evaluating the degree of contamination of Earth’s surface* (2016) (below abbreviated as Regulation of Evaluation of Contamination 2016).

## Results

### Emission of greenhouse gases to the atmosphere

Measurements of greenhouse gases emission from the settling pond for ashes and slugs did not reveal methane flux to the atmosphere at any sampling site. The flux of carbon dioxide at the reference K1 site was 14.4 g m^−2^ d^−1^ and changed from 7.3 to 42.1 g m^−2^ d^−1^ (Table 1) along the measurement line cutting through the dump area. Average carbon dioxide flux from the dump area (sites K5–K10) was 29 g m^−2^ d^−1^. Within the two zones (sites K4–K5 and K8–K10), the increased values of carbon dioxide emission were detected, in relation to the reference K1 site.

**Table 1:** Values of CO_2_ fluxes and atmospheric conditions recorded during measurements

Sampling point	Pressure (h Pa)	Air temp. (°C)	CO_2_ (ppm s^−1^)	CO_2_ flux (g m^−2^ d^−1^)
K-1	1002.7	10	0.90	14.4
K-2	1002.9	10	1.45	23.1
K-3	1002.4	10	0.95	15.2
K-4	1002.3	10	1.68	26.8
K-5	1001.4	10	2.11	33.6
K-6	1001.3	10	1.14	18.2
K-7	1001.4	10	0.96	15.3
K-8	1001.2	10	1.92	30.7
K-9	1001.2	10	2.64	42.1
K-10	1001.2	10	2.08	33.2
K-11	1002.1	10	0.45	7.3

### Spatial distribution of concentrations of selected elements in soils

In 33 soil samples, the following elements were analyzed: Cd, Cr, Cu, F, Mn, Ni, Pb, Ti, Zn, Li, Sr and V. Principal statistical parameters estimated for populations of these elements revealed that for Cd, Fe, Mn, Pb and Zn the mean concentrations were higher than their medians. Moreover, the populations of Cd, Pb and Zn concentrations showed higher standard deviation values than respective medians and means (Table 2), which may reflect the presence of anomalous values in these populations. Additionally, the mean concentrations of Cu, Fe, Mn, Ni, Pb, Zn, Li, Sr and V exceeded the background values estimated for soils in Poland (after Kabata-Pendias and Pendias 1993). Furthermore, the obtained analytical results were compared with the Regulation of Evaluation of Contamination (2016), which provides the permissible concentrations of particular elements for soil quality groups. In our study area, the soils belong to the Soil Quality Group III, which includes woodlands, shrubs, barren lands and miscellaneous lands. Generally, the average concentrations of analyzed elements did not exceed the values quoted for Soil Quality Group III, excluding Zn, which average concentration was higher than the permissible value by over 300 mg/kg (Table 2).

**Table 2:** Statistical parameters for selected elements detected in soil samples

Statistical parameters	Cd	Cr	Cu	Fe	Mn	Ni	Pb	Ti	Zn	Li	Sr	V
	(mg kg^−1^)											
Minimum	b.d.l.	23.2	22.8	15,086.2	299.2	17.8	7.8	142.1	63.5	13.4	21.9	24.3
Maximum	23.1	112.1	82.0	107,603.4	2616.3	49.3	1144.2	1187.9	8349.3	56.8	398.8	100.6
Average	5.8	46.7	52.5	42,434.6	901.0	36.4	193.4	739.4	1304.4	28.1	148.8	63.2
Median	4.5	44.6	52.8	29,003.9	761.3	35.6	60.1	783.4	337.9	25.6	147.5	63.5
Standard dev.	6.2	17.9	19.5	28,307.4	553.8	8.8	277.1	299.7	1993.1	11.7	100.1	20.7
Background (Poland)^a^	0.4	50	15	2800	500	25	25	3500	70	20	80	45
Limit value^b^	50	500	300	No data	No data	300	500	No data	1000	No data	No data	No data

*b.d.l.* below detection limit

^a^Background values estimated for Poland (after Kabata-Pendias and Pendias 1993)

^b^Limit value for group III grounds (after Regulation of Evaluation of Contamination 2016)

### Distribution of elements along the sampling line

The detailed analytical results for selected elements are presented as plots, which illustrate changes of concentrations along the sampling line for particular sampling depths. Additionally, the plots contain values recorded in reference samples from K1 site, 600 m away from the sampling line (Fig. 1) together with the background and the permissible values for particular elements provided by the above-mentioned Regulation of Evaluation of Contamination (2016).

**Fig. 3:**
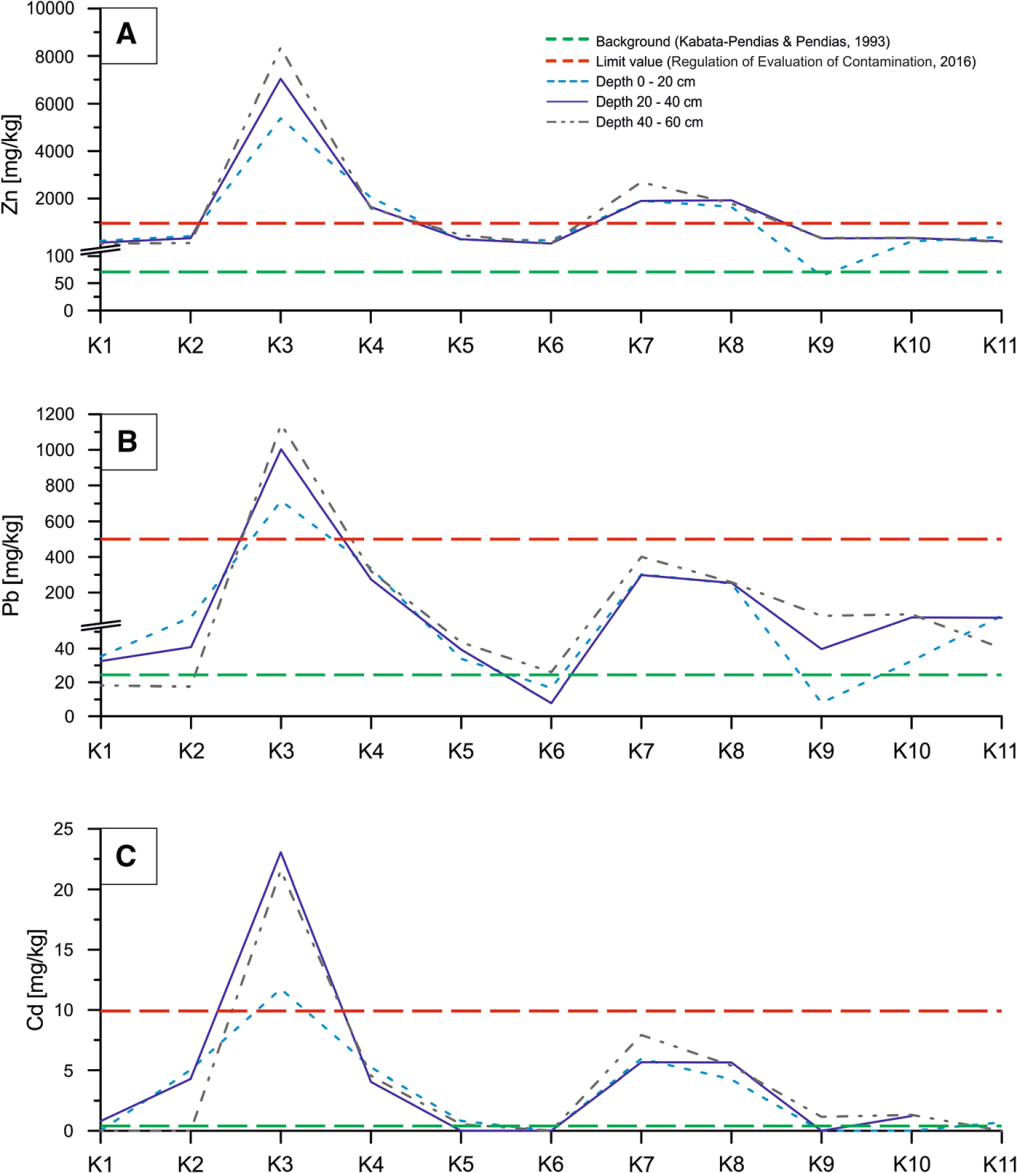
Concentration changes of Zn (**a**), Pb (**b**) and Cd (**c**) along profile

**Fig. 4:**
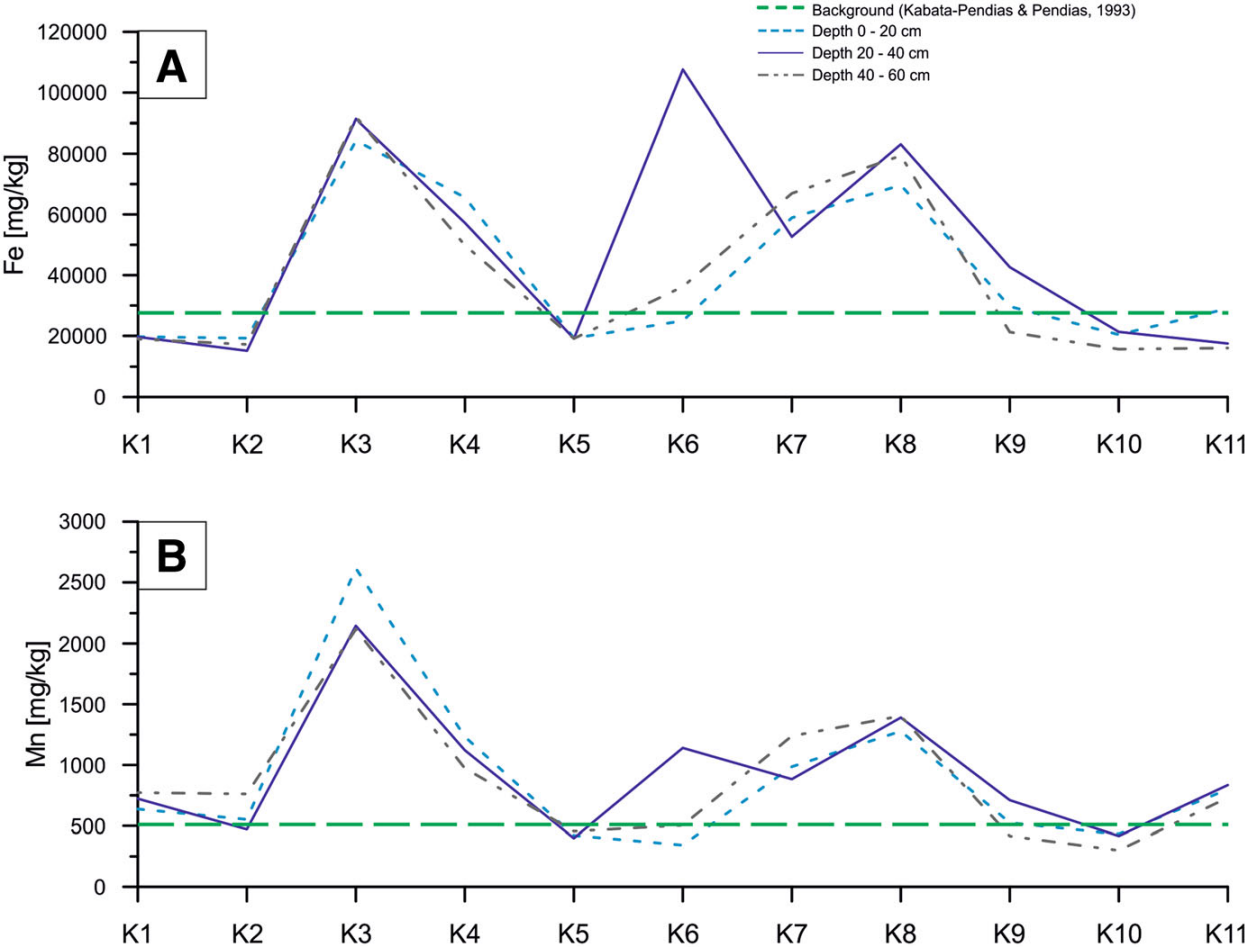
Concentration changes of Fe (**a**) and Mn (**b**) along profile

The plot of changes in Zn, Pb and Cd concentrations along the sampling line showed two anomalies at K3–K4 and K7–K8 sites, in which analyzed values exceeded both the background values for soils in Poland and the maximum values for Soil Quality Group III (Fig. 3). At the K3 sampling site, the concentrations of Zn, Pb and Cd were significantly higher than the permissible values quoted in the Regulation of Evaluation of Contamination (2016) at all three sampling depth intervals. Moreover, the permissible concentrations of Zn were exceeded also in samples from K7 and K8 sampling sites for all depth intervals (Fig. 3). Similarly to Zn and Pb concentrations, those of Fe and Mn formed two anomalies at K3–K4 and K7–K8 sampling sites where the background values for soils in Poland (after Kabata-Pendias and Pendias 1993) were significantly surpassed. Moreover, at the K6 and K9 sites, in 20–40-cm depth interval, the increase in Fe and Mn concentrations was evidenced in relation to values analyzed for the remaining sampling intervals (Fig. 4).

**Fig. 5:**
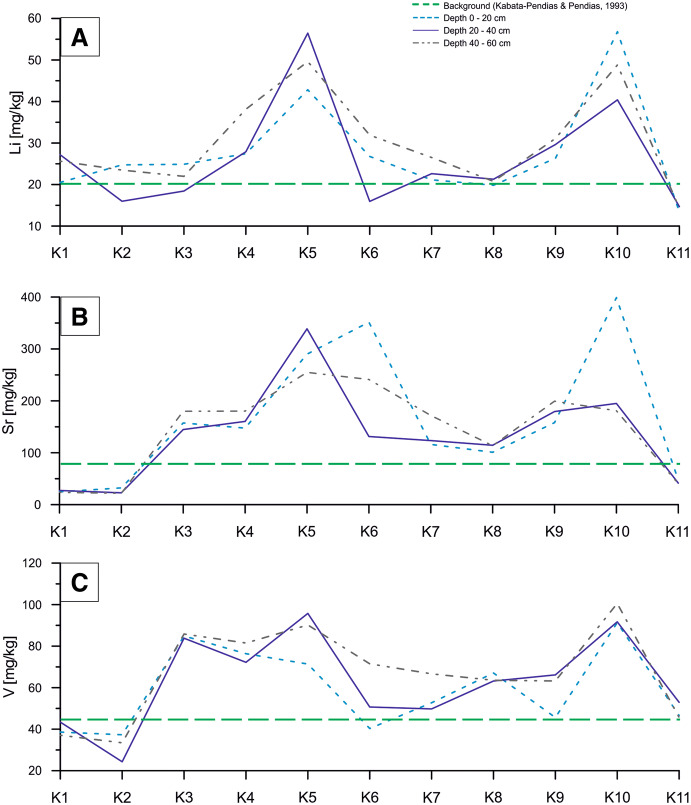
Concentration changes of Li (**a**), Sr (**b**) and V (**c**) along profile

The concentrations of Li, Sr and V revealed different patterns. Values exceeding the geochemical background for Li were found for K4, K5, K6 and K9–K10 sampling sites. For Sr, the increased concentrations were detected in the zones K3–K6 and K9–K10 of sampling sites. For V, increased concentrations were observed practically along the full length of the sampling line (from K3 to K10 sites) in 40–60-cm sampling interval (Fig. 5).

**Fig. 6:**
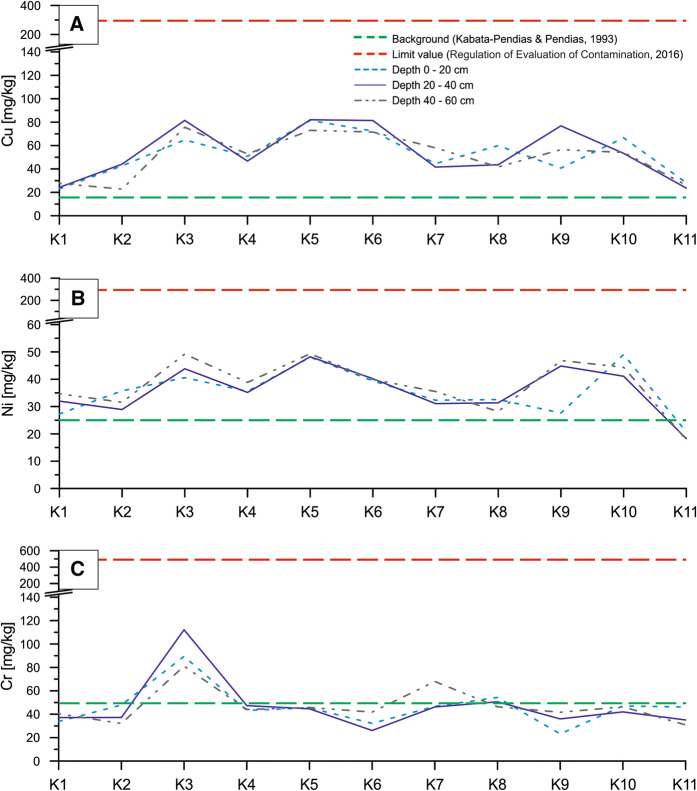
Concentration changes of Cu (**a**), Ni (**b**) and Cr (**c**) along profile

The concentrations of Ni and Cu along the sampling lines were quite uniform. Increased values were detected practically in all samples excluding those from the reference K1 site (600 m outside the sampling line) and from the end-line sites (K2 and K11) (Figs. 1, 6a, b). However, any analyzed sample did not reveal Ni and/or Cu concentrations over the permissible values quoted by the Regulation of Evaluation of Contamination (2016) (Fig. 6 A, B). Concentrations of Cr showed increased values only in samples from K3 site (in all sampling intervals), but they did not exceed the permissible value (Fig. 6c).

### Physical and chemical parameters of soils

In order to determine the principal physical and chemical features of analyzed soils, in each collected sample, pH, moisture and contents of organic matter, and carbonates were determined. Principal statistical parameters of obtained values are presented in Table 3. The pH values change from 7.23 to 9.57, the mean moisture content was about 20% with the maximum value of 61%, the organic matter contents varied from 2.5 to 29% and the carbonates contents changed from 0.7 to 11% (Table 3).

**Table 3:** Statistical parameters for physicochemical properties of soil samples

Statistical parameters	pH (average)	Moisture (%)	Organic matter (%)	Carbonate (%)
Minimum	7.23	6.7	2.5	0.7
Maximum	9.57	60.7	29.1	11.0
Average	8.48	19.3	9.3	2.8
Median	8.56	17.1	7.0	1.7
Standard dev.	0.51	11.5	7.0	2.7

**Fig. 7:**
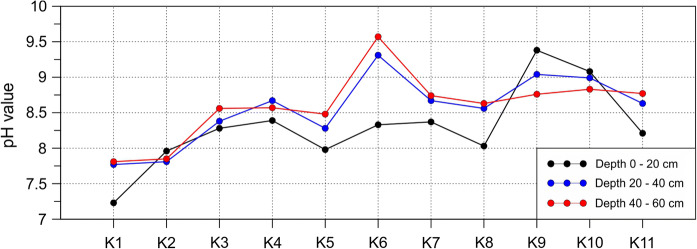
Changes of pH value along profile

Generally, the measured pH values increased with the depth. Such trend was observed in samples from eight sites (from 11), particularly at K3, K5 and K6 ones. Distinct drop of pH with the depth was found for soils from adjacent K9 and K10 sites (Fig. 7).

## Discussion

### Comparison of the results with concentrations of heavy metals in agricultural soils from Poland

The obtained concentrations of heavy metals in soils were compared with average concentrations in agricultural soils of the southern Poland (Terelak et al. 2000) and with concentrations in soil samples collected from local crop fields (Koncewicz-Baran and Gondek 2010).

The average Cd concentration in our study area is 5 times higher (Table 2) than average value quoted by Koncewicz-Baran and Gondek (2010). Additionally, in the topsoil interval of 0–20 cm, Cd concentrations far exceeding the average value for agricultural soils in this part of Poland were found in seven soil samples. In the K2 sample collected from a crop field at the foot of the settling pond, we detected very high Cd concentration in the topsoil—5.04 mg kg^−1^ (Fig. 3c). Even at the reference K1 site, distant by 600 m from the sampling line and the settling pond, Cd concentration in soil collected from depth interval of 20–40 cm was 0.8 mg kg^−1^, i.e., above the average value for soils from the southern Poland (Terelak et al. 2000).

The average Pb concentration for soil samples collected from the settling pond is 6 times higher than the average Pb concentration for agricultural soils in the southern Poland (Terelak et al. 2000; Koncewicz-Baran and Gondek 2010). Also, the samples from K1 and K2 sampling sites taken from the topsoil (0–20 cm) contain increased Pb concentrations in relation to the average value reported by Terelak et al. (2000) and Koncewicz-Baran and Gondek (2010).

In the study area, the average Zn value is two orders of magnitude higher (Table 2) than the concentration of Zn in agricultural soils from the southern Poland (Terelak et al. 2000) as well as at K1 and K2 sampling sites where Zn concentrations found in the top soil (0–20 cm) were much higher (217.3 and 413.1 mg kg^−1^, respectively) (Fig. 3a).

Similarly, average content of Cu in soil from the settling pond is over 5 times higher than average value for soils from the southern Poland (Terelak et al. 2000). In all soil samples collected from the topsoil 0–20 cm (including K1 and K2 sites), Cu concentrations are at least 2 times higher than average value for agricultural soils from the southern Poland (Terelak et al. 2000) and for local agricultural soils analyzed by Koncewicz-Baran and Gondek (2010).

The concentrations of other analyzed elements do not show such extreme differences in comparison with concentrations quoted for local agricultural soils by Koncewicz-Baran and Gondek (2010). The average Cr concentration in soil from the settling pond area is 46.7 mg kg^−1^, which is similar to average value (40.73 mg kg^−1^) found for agricultural soils from the southern Poland (Koncewicz-Baran and Gondek 2010). However, average Ni concentration in soil from the settling pond area is 2 times higher (Table 2) than the average value for soils from the southern Poland (Terelak et al. 2000).

Our results demonstrate that pollution of soils with heavy metals, especially Cd, Cu, Pb, Zn in the waste dumps and in neighboring areas, can be a cause for concern for local communities. In addition, any excess of local background values (Kabata-Pendias and Pendias 1993; Terelak et al. 2000; Koncewicz-Baran and Gondek 2010) and upper limit values for elements (Regulation of Evaluation of Contamination 2016) should be regularly monitored and subjected to detailed study.

### Impact of ashes with the addition of metals and carbonates to the soil

**Fig. 8:**
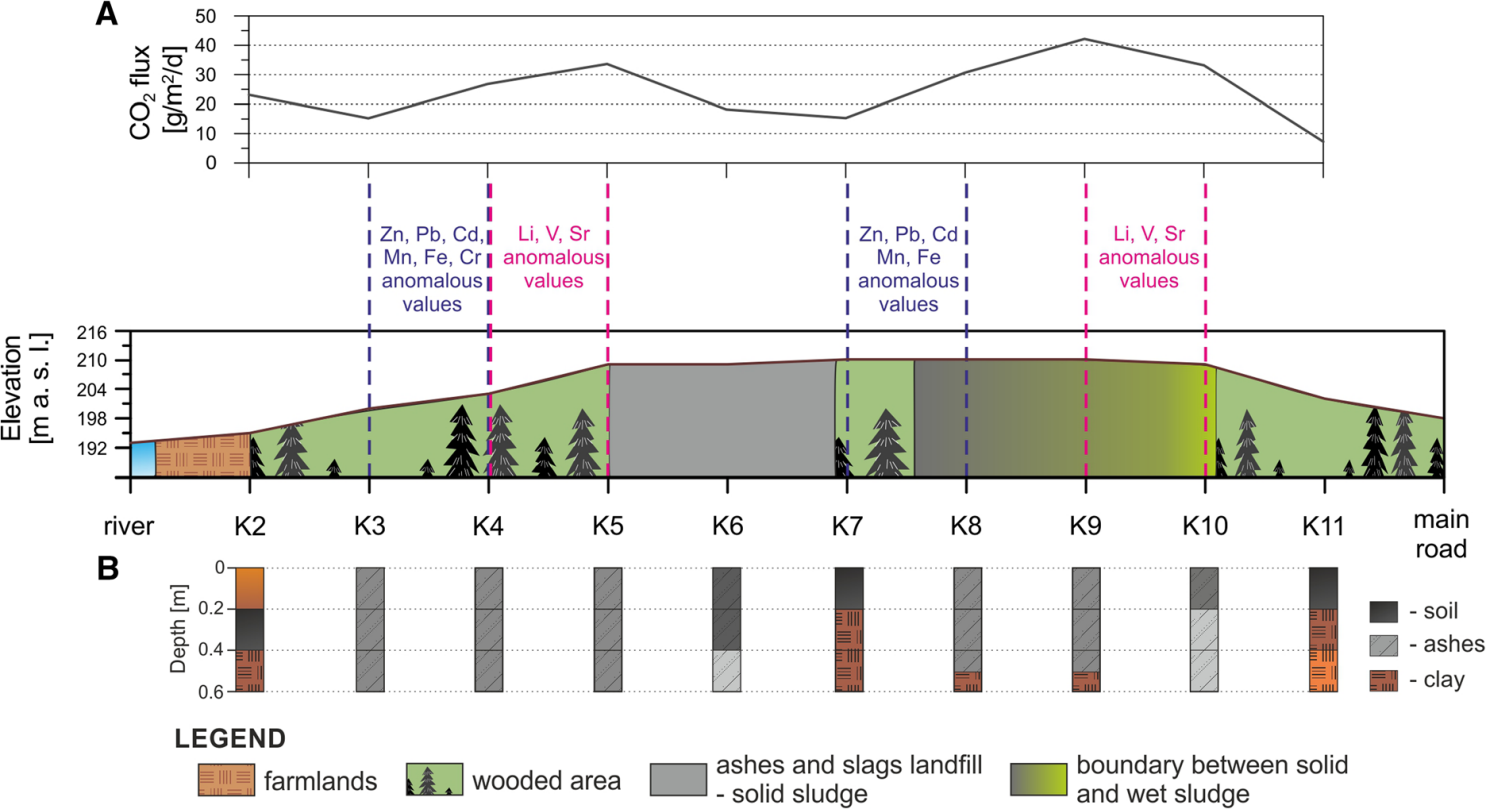
Diagrams showing the occurrence of anomalous concentrations of selected elements and values of CO_2_ fluxes along the cross section of the ash storage (**a**). Additionally, the **b** diagram shows change of subsurface sediments with depth

Soil from the study area was classified into the Soil Quality Group III, in accordance with the categories provided by the Regulation of Evaluation of Contamination (2016). The soil profiles from almost all sampling sites reveal the presence of ash (Fig. 8a). In samples from the forested and the woodland zones (K3, K4 and K5) located on the slope inclined toward the arable land and the riverside, ash was present along the full sampled soil profile (i.e., from the surface to 60 cm depth). Moreover, this soil interval hosts increased concentrations of trace elements: Zn, Pb, Cd, Mn, Fe, Cr, Li, V and Sr. The presence of ash in that part of soil profile suggests that in the past, this area was a part of settling pond, which subsequently dried and was vegetated by shrubs and trees (Fig. 8).

The recorded values of carbon dioxide flux do not differ significantly from natural values of that gas released from soils to the atmosphere in other regions of the world (Raich and Schlesinger 1992; Sanchez et al. 2002; Shi et al. 2006). The lack of methane together with relatively low carbon dioxide concentrations documents the negligible emission of biogenic gases from the dump (mixing layers of ash and hummus) to the atmosphere (Wysocka 2015). On the other hand, relatively increased carbon dioxide fluxes (over 30 g m^−2^ d^−1^) were detected in the two zones where anomalous values of heavy metals were recorded and the ash was present along with the full sampled soil profile (Fig. 8a, b). Obtained results may be related to the oxidation of carbon contained in metallurgical ashes (Fig. 8b). Previous studies showed that small amount of CO_2_ may be released from deposit of ashes (Heyer and Stegmann 1997) or as a residue of incineration of the organic carbon (Belevi et al. 1993).

Environmental studies carried out worldwide around smelters and mines revealed that ashes released from such plants may cause significant pollution of soil environment with heavy metals, mostly Zn, Cd and Pb (Pruvot et al. 2006; Douay et al. 2008; Shukurov et al. 2014). According to Pruvot et al. (2006), maximum concentrations of Cd and Pb in 0–25-cm depth interval were 31.1 and 3711 mg kg^−1^, respectively, whereas the research run in the northern France around two zinc and lead smelters revealed the following concentrations of trace elements in soil samples collected from depth interval 0–25 cm: for Cd—from 3.1 to 31.4 mg kg^−1^, for Pb—from 95 to 3711 mg kg^−1^ and for Zn—from 326 to 6908 mg kg^−1^ (Douay et al. 2008). Similar studies carried out in the industrial region of the northwestern Uzbekistan demonstrated the pollution of upper soil horizon with Cd, Pb and Zn in the vicinity of a smelter. The maximum concentrations of these elements were: Cd—30 mg kg^−1^, Pb—630 mg kg^−1^ and Zn—3010 mg kg^−1^ (Shukurov et al. 2014). Studies on pollution around the smelters worldwide showed maximum Cd concentrations in soils about 30 mg kg^−1^, whereas in our study area, maximum Cd concentration detected in 20–40-cm depth interval was 23 mg kg^−1^ (Table 2, Fig. 3c), i.e., it was at the same order of magnitude that values are obtained around smelters worldwide. In the study area, concentrations of Pb in samples from the vicinity of settling pond reached up to 1144 mg kg^−1^ (Table 2, Fig. 3b), which also corresponds to lead concentrations in the above-mentioned regions of metallurgical industry in the world. However, maximum Zn concentration detected in our study area (8349 mg kg^−1^, Table 2, Fig. 3a) is significantly higher than values provided for both the northern France and the northwestern Uzbekistan (Douay et al. 2008; Shukurov et al. 2014). Additionally, such concentration exceeds 8 times the permissible value for Soil Quality Group III provided by the Regulation of Evaluation of Contamination (2016).

Such results indicate that heavy metals (including Cd, Pb and Zn) are contained not only in the ashes released to the atmosphere, as shown by above-mentioned publications (Pruvot et al. 2006; Douay et al. 2008; Shukurov et al. 2014) but occur also in other wastes deposited in the settling pond.

### Leaching of metals and impact of reforestation

The slope inclination may cause migration of leachates from the central part of the dump toward the river, and scarce vegetation may facilitate intensive wind erosion, and airborne transport of wastes to the adjacent land (Wysocka 2015). Such opinion is supported by distribution of Zn, Pb, Ni, Fe and Li concentrations at K2 sampling site located in a crop field (Figs. 3a, b, 4a, 5a, 6b). The highest concentrations of these elements were detected in the topsoil (0–20 cm), which proves that any agricultural activity in the vicinity of the dump should be banned. The K6 sampling site was placed directly on the surface of ash dump, as confirmed by the alkaline pH of analyzed samples—at that site the highest pH value was measured (Fig. 7). Moreover, that site did not show anomalous amounts of most of the analyzed elements except for the increased Fe and Mn concentrations detected at 20–40-cm depth interval and the increased Sr concentrations found in the topsoil (0–20 cm) (Figs. 4, 5). This may be the result of flat relief at the site and/or the tendency of pollutants to migrate as leachates to dump interior and along its slopes (Wysocka 2015), as confirmed by the anomalies disclosed on both sides of the central part of the dump (Fig. 8). Moreover, pollutants migrating down the dump may then percolate to groundwaters and further, to small surface flows, and to the river (Jensen et al. 2000; Bakis and Tuncan 2011; Migaszewski and Galuszka 2016; Gwenzi et al. 2016; Han et al. 2016; Xu et al. 2018; Söderberg et al. 2019).

Our results demonstrate that pollution of soils with heavy metals may also be caused by: (1) wind erosion of ashes from metallurgical waste dumps and their airborne transport to the surrounding crop fields as well as by (2) potential migration of unwanted elements to soil horizons and groundwaters, and further, to surface flows.

Earlier studies show that the impact of smelter ashes on soil and plant environments is negative (Pruvot et al. 2006; Douay et al. 2008; Shukurov et al. 2014). According to Pruvot et al. (2006), heavy metals in soils, dust and plants may be potentially mobile and the local community can be the exposure to the pollution by inhalation, direct ingestion of particles and consumption of plants (Pruvot et al. 2006). Douay et al. (2008) also paid attention to the possibility of metal penetration into deeper layers and transfer heavy metals to the food chain. Large amounts of heavy metals, especially Cd and Pb, in the plant environment can cause growth inhibition, reduction of photosynthesis, changes in the activity of some enzymes. In addition, anomalous accumulations of Pb and Cd in human organisms can pose serious health problems such as inhibition of growth rate, anemia, encephalopathy, kidney damage and disorders of the nervous system (Węglarzy 2007).

Authors of this publication believe that reforestation of central part of waste dump can contribute to stop the processes of wind erosion and limit the transfer of ashes with heavy metals to adjacent arable land. Furthermore, several different phytoremediation technologies are well known in which trees are used for decontaminate soil polluted by heavy metals (Liu et al. 2013). Planting appropriate species of trees in the central part of the waste dump could stop the wind erosion processes and help purify the soil of heavy metals.

### Physical and chemical properties of elements in soil

The physical and chemical properties of soils significantly affect the mobility of trace elements. Water is one of main factors controlling the mobility of elements in soil by changing the soil pH to more acidic (Nowińska and Adamczyk 2013). Intensification of metal transport in soil can occur during rain periods. In some environmental pH of water, rain varies from 6 to 7. Additionally, in the southern Poland extremely low pH values of water rain were recorded in the range of 3.5–6.5 (Nowińska and Adamczyk 2013).

In our samples, pH values varied from 7.23 to 9.57 which indicate slightly alkaline to alkaline conditions. Such conditions favor the immobilization of metals in soil. However, in the event of a rapid change in the pH caused, for example, by pouring a nearby river (flood), accumulated metals can be activated and become more mobile in the environment. In addition, acid rain may also change the pH of the solution to more acidic.

Depending on the chemical properties, the particles have different mobilities in the environment. When the pH changes to more acidic, Zn and Cd will be activated first (pH < 6–6.5) and then Pb and Cu (pH < 5) (Nowińska and Adamczyk 2013).

Another factor immobilizing trace metals in soil may be the high concentrations of Fe and Mn. In the soil, Fe and Mn oxides have a high sorption capacity which may control the mobility and bioavailability of heavy metal pollutants. The Fe and Mn compounds have a high surface area and high surface charge density what is used to remove the heavy metals from soil fines (Gasparatos 2013). Coexistence of large amounts of Fe and Mn with anomalous concentrations of metals (Pb, Cd, Zn, Cr) that occur in the waste dump may indicate that metals are immobilized (Fig. 8a). This correlation is clearly visible in the following sampling points: K3, K4, K7 and K8 (Figs. 3, 4, 6, 8a).

Further research should focus on determining the mobility and bioavailability of individual trace elements (Cd, Cr, Pb) whose concentrations exceed the assumed threshold values and may pose a potential risk to the environment of adjacent areas and human health.

## Summary and conclusions

Geochemical studies that run in the area of industrial wastes dump localized within an urban area in the southern Poland reveal that:

- total concentrations of heavy metals in analyzed soil samples highly exceed permissible values for Cd, Pb and Zn in soils classified into the Soil Quality Group III, quoted by the Regulation of Evaluation of Contamination (2016),
- concentrations of Cd, Cu, Zn, Pb, Fe, Mn, Ni, Li, Sr and V in analyzed soil samples exceed natural background values,
- in the vicinity of waste dump, concentrations of Pb, Zn, Cd and Cu significantly (several times) exceed both the natural background values and the values typical of local agricultural soils from southern Poland,
- high accumulation of heavy metals in soils from the vicinity of waste dump may result from improper protection of land onto which the dump has been developed in the past,
- in the future, detected high pollution of soils with heavy metals may generate significant environmental hazard because changes of soil environment (e.g., evolution of pH toward more acid conditions) may increase mobility of elements recently stabilized in the soil, which, in turn, may cause serious threat to living organisms.

The preliminary study of industrial waste dump proved that further research is necessary in order to evaluate the quality of soils, particularly in the areas surrounding this object. Such research should include the possible leaching of analyzed heavy metals and their migration to groundwaters and to surface flows (among others to adjacent river) because such migration may generate serious hazard for aquatic environment and for vegetation cover. Moreover, a health risk assessment should be undertaken due to agricultural activity carried on by local residents around the dump and resulting potential accumulation of heavy metals in produced crops, which may cause threat to human’s health and life.
